# Developing and Implementing All-in-One Standard Paediatric Parenteral Nutrition

**DOI:** 10.3390/nu5062006

**Published:** 2013-06-05

**Authors:** Rosan Meyer, Meike Timmermann, Sven Schulzke, Caroline Kiss, Marc A. Sidler, Raoul I. Furlano

**Affiliations:** 1 Department Gastroenterology, Great Ormond Street Hospital Foundation Trust, London, WC1N 3JH, UK; 2 Hospital Pharmacy, University Hospital Basel, Basel, 4031, Switzerland; E-Mail: mtimmermann@uhbs.ch; 3 Department Gastroenterology and Nutrition, University Children’s Hospital Basel (UKBB), University of Basel, Basel, 4056, Switzerland; E-Mails: sven.schulzke@unibas.ch (S.S.); marc.sidler@hin.ch (M.A.S.); raoul.furlano@ukbb.ch (R.I.F.); 4 Department Nutrition and Dietetics, University Hospital Basel, Basel, 4031, Switzerland; E-Mail: kissc@usb.ch

**Keywords:** parenteral nutrition, all-in-one parenteral nutrition, paediatrics, development, implementation

## Abstract

Parenteral nutrition (PN) is a feeding mode suitable for children that do not achieve requirements via the enteral route. For this intervention to be successful, healthcare professionals require: knowledge on nutrient requirements; access to an aseptic compounding facility; and a system that ensures adequate and safe delivery of PN. Previously, it was thought that individualised PN was the “gold standard” for delivering nutrients to children; however, studies have highlighted concerns regarding inadequate delivery of nutrients, prescribing and compounding errors. We, therefore, set out to develop and implement all-in-one (AIO) paediatric PN solutions. Through a systematic approach, four AIO PN solutions were developed: birth–two months of age (Ped 1); two months–10 kg (Ped 2); 11–15 kg (Ped 3); and 16–30 kg (Ped 4). We implemented them with the help of a teaching pack, over a one month time period, and reviewed usage at six months. At that time, five children initially received standard PN without electrolyte changes; but after a few days, electrolytes needed amendments, and three required individualised PN. A change to AIO PN is feasible and safe; however, some may require electrolyte changes, and there will always be those that will require individualised PN.

## 1. Introduction

Parenteral nutrition (PN) is a feeding mode suitable for infants and children that cannot be fully fed via the enteral route [1]. This form of nutritional support can transform the outlook for patients that do not have the ability to achieve nutrient requirements via the enteral route, due to acute or chronic intestinal failure [2,3]. However, for this intervention to be successful in paediatrics, healthcare professionals require: knowledge on macro- and micro-nutrient requirements; access to an aseptic compounding facility; and finally, access to a system that ensures adequate and safe delivery of PN to the patient [4,5]. In the past, individualised PN was thought to be the “gold standard” for achieving optimal nutrient intake and patient safety. However, several studies have highlighted serious prescription, as well as compounding, errors and have identified the ordering and manufacturing of PN as a high risk activity [4,5,6,7,8]. A study by Brown *et al*. [6] found prescription errors in 27.9% of neonatal PN, and a separate study found that 54.1% were inadequately assessed to predict nutrient requirements [8]. As a result, there has been a move towards standardisation of PN for both neonates and paediatrics [9]. The use of standard PN has been shown to increase pharmacy aseptic manufacturing capacity, reduce electrolyte imbalances and lead to improved delivery of nutrients, when compared to individualised bags [7,10,11]. A study by Klüttgens *et al*. [12] in 2003 found that 17% of paediatric units in Europe used standard PN, and Bouchard *et al*. [13] repeated this study in 2009 and found that 43% of paediatric/neonatal centres in European hospitals had used some form of PN standardisation [12,13]. However, both studies indicated that the practice of using standard PN was significantly more common in neonatal units. Unlike adult PN, not many commercial paediatric standard solutions are available, and numerous specialist paediatric centres have resorted to developing their own standard PN. However, of existing standard solutions, the majority provide only the aqueous solution of amino acids and glucose, and lipids are required to be given separately. We therefore set out to develop and implement all-in-one (AIO) paediatric PN solutions for a paediatric hospital and to describe the process and our experience with the implementation.

## 2. Methodology

### 2.1. Developing the Composition of the Standard PN

The new standard PN bags were developed for the University Children’s Hospital Basel (UKBB), in Switzerland. This hospital annually admits 7000 children with a variety of diagnoses, including surgical and gastrointestinal conditions that may require short- or long-term PN. No standard PN practice was in place at the time that this project was started, and the normal practice was to use adult standard PN solutions for children or to order individualised PN. Retrospective data from the hospital indicated that, in 2009–2010, the hospital utilised 103 adult-type PN AIO bags for paediatrics.

Published guidelines from the European Society of Paediatric Gastroenterology Hepatology and Nutrition (ESPGHAN) from 2005 were used as baseline for the development of the standard PN [1]. In addition, composition data from any existing age-appropriate standard bags, produced by commercial companies, were reviewed. As advances had been made in composition of PN since the publication of the ESPGHAN guidelines, we also performed a literature search on PubMed-NCBI, to ensure the inclusion of new data related to PN, using the following search terms: paediatric parenteral nutrition; standard parenteral nutrition; parenteral lipids; parenteral amino acids; parenteral carbohydrates; and parenteral vitamins/minerals in paediatrics. This literature search pointed towards a shift in the use of lipid emulsions, due to documented side effects with first generation PN lipids based on soya phospholipids [14]. In particular, in children with short bowel syndrome with PN associated liver disease [15,16,17], studies have found a positive impact with lipid solutions containing omega-3-fatty acids (fish oil), and a reduction in oxidative stress was also seen in neonates on PN [15,17]. We, therefore, made the decision to use SMOF lipids (Fresensius Kabi, Oberdorf, Switzerland) as our standard fat solution. 

Following the gathering of information, the specialist PN pharmacist and dietician produced suggested PN compositions that complied with the ESPGHAN guidelines. These compositions also complied with Safe Practice Guidelines for Parenteral Nutrition [5] for compounding, using the macro- and micro-nutrient solutions in Table 1. A decision was also made to add a standard amount of vitamins and minerals to the AIO PN bags, because prescription errors were common with micronutrients in our hospital, and to discourage any additions at the ward level. 

**Table 1:** Macro- and micro-nutrients used for the all-in-one (AIO) standard solutions.

Component	Name	Manufacturer
Amino acids	Aminoven Infant 10%	Fresenius Kabi, Oberdorf, Switzerland
Glucose	Glucose 50%	Grosse Apotheke Dr. Bichsel AG, Interlaken, Switzerland
Lipids	SMOF Lipid	Fresenius Kabi, Oberdorf, Switzerland
Vitamins	Soluvit N	Fresenius Kabi, Oberdorf, Switzerland
	Vitalipid N Infant	Fresenius Kabi, Oberdorf, Switzerland
Trace Elements	Peditrace	Fresenius Kabi, Oberdorf, Switzerland
Sodium	Sodiumchloride (Concentration 59 mg/mL)	Manufactured in the hospital pharmacy
	Sodiumacetate (Concentration 1 mmol/mL)	Manufactured in the hospital pharmacy
Potassium	Potassiumchloride 7.45%	Sintetica, Mendrisio, Switzerland
Magnesium	Magnesiumchloride 0.5 M	B. Braun, Sempach, Switzerland
Phosphate	Glycophos	Fresenius Kabi, Oberdorf, Switzerland
Calcium	Calcium Sandoz 10%	Sandoz Pharmaceuticals, Novartis, Switzerland

The suggested AIO PN solutions were then assessed by physicians and nursing staff from different medical disciplines, including: gastroenterology; neonatology; intensive care; oncology and surgery. Each speciality assessed the AIO PN for nutrient adequacy and practicality in their respective populations. The PN composition was subsequently amended with further input from the dietician and pharmacist. 

Once the composition of the PN bags were finalised, they were sent to Fresenius Kabi (Bad Homburg, Germany) for stability testing. Stability tests for the lipid and aqueous phase, as well as admixtures, were performed, including: pH, particle droplet size and distribution, appearance, discolouration, turbidity and outer limits of additions (*i.e.*, divalent cations, such as calcium and magnesium).

### 2.2. Implementation of the Standard PN

**Figure 1:**
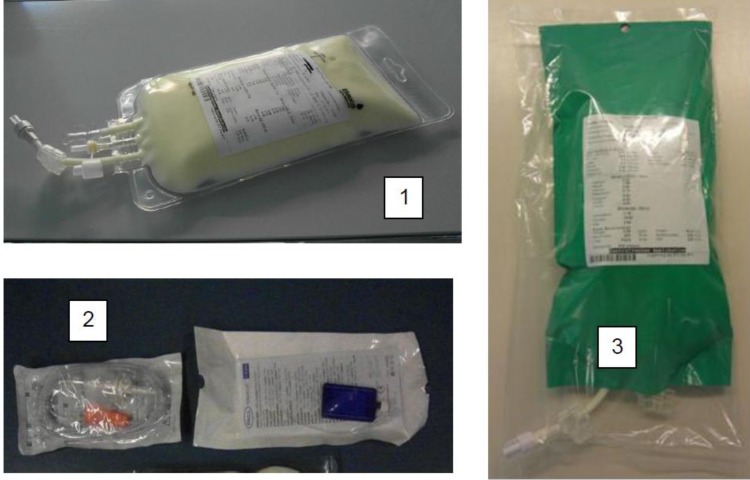
Illustration on PN solutions and how they were delivered to the ward: (1) is the AIO PN bag; (2) the giving sets and filter used and (3) the light protection and bar coded label for a standard PN solution.

Prior to implementation of the AIO standard PN, a risk analysis of the process (*i.e.*, prescribing, compounding, label production and quality control) was performed to limit errors. An electronic ordering system was put in place that provided reference nutrient requirements for each age group to guide physicians [1]. All nutrient calculations were completed automatically, resulting in no prescription errors. At the pharmacy level, all prescriptions were reviewed, and an electronic warning system was in place if any electrolyte additions exceeded safe levels set out by the ESPGHAN guidelines. This would trigger a phone call to the prescribing doctor, to ensure the prescription was correct. Compounding occurred in the hospital pharmacy using the Baxa Compounder EM2400 (Baxter, Engelwood, CO, USA) according to the current Safe Practice Guidelines [5]. Volumetric delivery was checked gravimetrically (accuracy ±3% for volumes >4 mL), and final checks (visual control of PN bag, production record) were performed by a pharmacist. The PN was delivered in ethylene-vinyl acetate (EVA) bags and covered to protect against ambient light to prevent oxidation of micronutrients and peroxidation of lipids (Figure 1).

**Figure 2:**
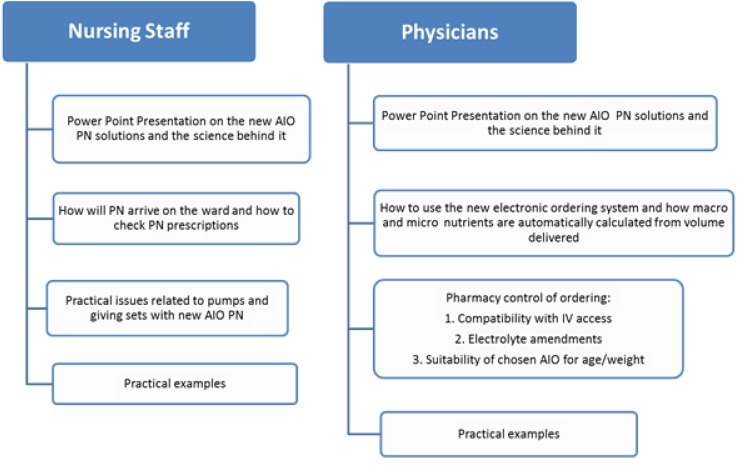
Information provided during teaching for physicians and nursing staff.

The implementation of the new AIO was conducted by the project lead dietician and pharmacist and involved two separate teaching packs; one for nurses and one for physicians (Figure 2). As physicians were allowed to amend electrolytes on standard solutions for individual patients, as well as vitamins and minerals for long-stay patients, specific guidance was given on this topic to physicians. In addition, the newly developed PN protocol was available electronically as a reference guide for healthcare professionals. After teaching was completed, one month was allowed to implement the new standard PN system into the daily workflow of the paediatric unit. During this time, both the dietician and pharmacist, who led the development of the new AIO PN bags, were available daily. They also liaised with ward physicians who ordered the PN, as well as with nursing staff, who were responsible for attaching the PN to the patient.

### 2.3. Review of Usage

After the introduction of the AIO PN (February 2011), we evaluated the usage of the standard solutions for 6 months (June–December 2011), in the following way:

1. How many were ordered?
2. How many children required electrolyte corrections?
3. How many children required an individual PN solution?
4. If any ordering/compounding errors were made.

## 3. Results

### 3.1. Composition

Information on the composition of existing standard PN solutions were obtained from paediatric units in Switzerland (two-bag system), South Africa (AIO), United Kingdom (two-bag system), Australia (two-bag system), as well as a company-manufactured two-bag standard bag system (Fresenius Kabi, Runcorn, UK). Information was also sourced from Krohn *et al.* [18], who published data on eight standard solutions used in the intensive care unit and amended nutrient compositions and constituents according to new research that had followed ESPGHAN guidelines in 2005. As a result, four AIO standard PN solutions (Table 2), based on a combination of weight and age of children, were developed: Ped 1, which was suitable from birth (3 kg) until two months of age (1000 mL); Ped 2, from the age of two months to 10 kg (1000 mL); Ped 3, from 11 to 16 kg (1500 mL); Ped 4, from 16 to 30 kg (2000 mL). Children above 30 kg used the adult standard solutions. The content of the standard PN solutions were developed that, if delivered in normal expected volumes, the recommended amount for macro- and micro-nutrients based on ESPGHAN guidelines were achieved (Example 1).

**Table 2:** Four AIO parenteral nutrition (PN) solutions.

	Ped 1	Ped 2	Ped 3	Ped 4
	Birth–2 months	2 months–10 kg	11–16 kg	16–30 kg
	(1000 mL)	(1000 mL)	(1500 mL)	(2000 mL)
	per 100 mL	per 100 mL	per 100 mL	per 100 mL
*Amino Acid (g)*	2.19	2.5	2.67	3.38
*Glucose (g)*	10.5	14.5	14.47	14
*Lipids (g)*	2.5	2.8	3	3.5
*Energy (kcal)*	67	85	87	93
*Sodium (mmol)*	2.2	2.5	3	3.5
*Potassium (mmol)*	1.48	2	2.53	3.3
*Magnesium (mmol)*	0.15	0.2	0.19	0.18
*Calcium (mmol)*	0.5	0.8	0.25	0.3
*Phosphate (mmol)*	0.5	0.8	0.25	0.3
*Chloride (mmol)*	1.8	2.42	3.92	5.12
*Acetate (mmol)*	1.19	0.89	1.5	1.45
*Vitalipid/Soluvit 1:1 (mL)*	1.92	1.33	0.85	0.7
*8Peditrace (mL)*	1	1	1	1
*Osmolarity (mOsmol/L)*	907	1185	1210	1280

**Example 1:** Delivery of nutrients using Ped 3 for an 18 month toddler weighing 12 kg. ESPGHAN, European Society of Paediatric Gastroenterology Hepatology and Nutrition.

	ESPGHAN requirements [1]	Nutrients delivered
*Fluid (mL/kg)*	80–120	90
*Protein (g)*	1.5–2.5	2.4
*Glucose (g)*	12–14	13
*Lipids (g)*	2–3	2.7
*Energy (kcal)*	75–90	86
*Sodium (mmol)*	2–3	2.7
*Potassium (mmol)*	1–3	2.28
*Magnesium (mmol)*	0.2	0.17
*Calcium (mmol)*	0.2	0.23
*Phosphate (mmol)*	0.2	0.23
*Vitalipid/Soluvit 1:1 (mL)*	10	9
*Peditrace (mL)*	15	9

Stability testing indicated that our PN solutions with additives were stable for six days at a temperature of 2–8 °C and one day at room temperature. During the six month period, no compounding errors or other stability problems were experienced with the AIO PN. 

### 3.2. Data on Ordering of Standard PN

No prescription errors were made during the six month observation period. Data on PN prescriptions indicated that during this time, eight children required PN. Five of the eight children were initially commenced on AIO PN (total of 36 PN bags) with no electrolyte changes; however, subsequently, all of these five children required electrolyte amendments, (total of 64 PN bags), and three children needed individual PN from the start (Table 3). 

**Table 3:** Patient characteristics.

	AIO PN with/without electrolyte changes	Individual PN
Number of children	5	3
Age in years (median)	2.2 (range 0.8–5)	12.6 (range 7–17)
Gender	2 male	3 male
Diagnosis	4 short gut1 post gastrointestinal surgery	3 graft *versus* host disease post bone marrow transplantation
Electrolyte amendments/reason for individual PN	4 required amended potassium1 required the removing of sodium acetate	All 3 had hyperglycaemia, hyperlipidaemia and significant electrolyte disturbances.

After six months, we also compared the cost impact of the standard PN solutions against individual PN. The direct cost for the Ped 1 and Ped 2 standard bags were similar for the individual PN solutions (Ped 1 90.48 Euros and Ped 2 94.63 Euros); however, this excluded indirect cost related to staffing, time and a reduction in potential errors. The cost for Ped 3 and Ped 4 was 112 Euros and 166.04 Euros, respectively, as compared to 116.23 Euros and 186 Euros for individual PN solutions of similar volume and also excluded indirect cost.

## 4. Discussion

Although AIO PN solutions have recently been introduced in preterm infants [19], this is, to the knowledge of the authors of the first publication, describing the process of developing and implementing AIO standard PN in a paediatric setting. The implementation of standard PN solutions in our hospital occurred, due to an absence of a standardised PN process in the unit and a general move towards standard PN solutions in paediatrics, as a result of studies highlighting substandard individualised PN practice [6,18,20,21]. In 2007, the American Society for Parenteral and Enteral Nutrition (ASPEN) suggested a standardised process for PN management, which included the implementation of standardised PN formulations [7], which are very much in line with the more recent paediatric recommendations by Fusch *et al*. [9] for short-term PN. However, the ASPEN guidelines also recommend the availability of a mechanism for compounding individual PN for patients that fall outside of the remits of standard solutions. All children in this study were initially started on AIO PN, but due to the underlying diagnosis (Table 3), eventually required electrolyte amendments, and three required individualised PN. In the literature, it is acknowledged that there will be children that do not fit into a standard regime. In our study, 23% (*n* = 3) required individualised PN, whereas Porras *et al.* [20] found that 17% of children, in their general paediatric setting, needed individualised PN. Krohn *et al.* [18] reported that 32% of their cohort on the paediatric intensive care unit required individual PN. The difference in the number of individualised PN required between different studies can easily be explained by the patient diagnosis and setting. All three patients requiring individualised PN in our study were post-bone marrow-transplant with graft *versus* host disease and required long-term PN. The use of individualised PN in these patients was appropriate, as none of our standard solutions could provide macro- and micro-nutrients unique to this diagnosis. According to the European Society for Parenteral and Enteral Nutrition (ESPEN) [22], individualised solutions may be preferable for non-surgical oncology patients requiring long-term PN.

In this study, all five patients required standard PN with electrolyte changes at some point, with the majority requesting changes in potassium. Porras *et al.* [20] reported that only 4% of their cohort required electrolyte changes, *versus* 27.5% electrolyte modifications in the paediatric intensive care cohort receiving full PN [18]. The number of children requiring electrolyte changes in our study is significantly higher, due to the particular patient cohort with short bowel syndrome, a well-known complication in children with this disorder [23]. Also, in our experience, a number of electrolyte changes were requested to correct acute metabolic derangements. Muehlebach *et al.* [24] discussed handling of AIO PN, recommending that acute electrolyte corrections should ideally not be done through standard PN. In our hospital, prior to the introduction of standard solutions, additions were often made at ward level, which have been highlighted in publications as introducing dosage errors, bacterial contamination and may also impact on the stability of the PN [12]. In our teaching pack, we did not specifically address acute electrolyte imbalances and how, ideally, these should be addressed in children receiving AIO PN. However, other centres embarking on implementing standard PN should address these issues with physicians prior to implementation. They should avoid using standard PN for making acute electrolyte changes and, ideally, not make any additions to the PN at ward level. 

The composition of our four AIO PN bags yielded good levels of nutrients when optimal fluid volumes were prescribed, as recommended by ESPGHAN [1]. In those children who are fluid restricted (*i.e.*, cardiac surgery), this may be perceived as a problem. However, if long-term PN was required in such patients, an individualised PN solution was recommended by our protocol. Due to the potential significant variation in body weight, it is often thought that standard PN solutions may be more suitable, as suggested by Krohn *et al*. [18], who had eight standard PN solutions for their patients in the paediatric intensive care unit. However, in our experience, four standard PN solutions were adequate for the needs of the type of patients requiring PN in our hospital. This is similar to Porras *et al.* [20], who found that three standard PN solutions were sufficient for their paediatric population. The latter study found that energy requirements were met within three days of patients receiving standard PN, whereas the individualised PN had an average deficit of 33% in energy. Although we had guidelines in place for physicians to advance the PN in order to achieve requirements within 3–4 days, depending on baseline nutritional status, we did not assess the speed of reaching requirements. This was due to the low number of children receiving PN over six months. This is a limitation of this study. However, future prospective studies in our institution are planned to evaluate this, once sufficient patients have received the standard PN. 

The choice of lipids (*i.e.*, SMOF lipids) may be criticised following the recent systematic review on lipid emulsions containing omega-3-fatty acids. That study cited three high quality studies and five randomised controlled studies and found that there was insufficient evidence to use lipid emulsions with omega-3-fatty acids for children with intestinal failure and other conditions [25]. This result is possibly related to the low number of studies and the variation in study design. At the time of developing the AIO PN, this systematic review was not available, but we made our choice based on the documented negative side-effects related to first generation lipid, the closer resemblance of SMOF lipids to what would have been orally consumed by infants and data hinting towards a reduced risk of developing PN-related liver disease [15,16,17,26].

The addition of vitamins and minerals as standard into our AIO solution may also be perceived as a limitation. However, we resorted to this practice, due to errors previously identified in our hospital. Skaourliakou *et al.* [27] investigated physiochemical stability of AIO standard neonatal bags and found that the stability of admixtures are very much dependant on the content of α-tocopherol. In addition, a recent publication by Wong *et al.* [28] highlighted the lack of evidence for current recommendations for trace elements in PN for children and advised against using AIO trace element solutions (*i.e.*, Peditrace, Fresenius Kabi). Instead, they recommended the use of individualised trace element supplementation. Although there is a paucity of data, this is practically very difficult and potentially very cost-ineffective. We found that by having standard amounts of micronutrients in the PN, we were assured that all children received sufficient vitamins and minerals. We recommended that in long-term PN, blood values for both vitamins and minerals are assessed and adjusted accordingly on an individual basis.

Finally, assessing the process of PN from prescription to delivery, as suggested by Bonnabry *et al.* [4], was an extremely useful exercise and enabled us to establish the risk in error. The change to AIO PN solutions would not have been possible if we had not also switched onto an electronic ordering system, a new labelling system, changed the compounder and the method of delivery of the PN. Keady *et al.* [29] found that with the use of standard PN, aseptic capacity in pharmacy can be significantly increased, which concurs with our experience. We also found that the direct cost for manufacturing standard solutions were similar or reduced, when compared to individualised PN. Gamsjaeger *et al.* [30] compared the actual cost of individualised PN *versus* standard PN and found that, on average, individualised PN per day cost 82.78 Euros, *versus* 61.21 Euros. In our unit, there were concerns related to the wastage of volume in standard PN. However, this did not have a cost implication, as indicated by our cost analysis, and in particular, the Ped 3 and Ped 4 were less costly than individualised PN. Future studies into the cost benefit should take the cost related to potential error reduction with standard PN also into account. 

## 5. Conclusions

Four AIO standard PN solutions, with a stability of seven days, were successfully developed and implemented in a paediatric hospital. Both development and implementation required the input of a multidisciplinary team that analysed the process of PN management to reduce errors. Our study shows that not all children will have their requirements met with standard PN. For those patients, the capacity to make amendments to standard solutions and compound individualised PN needs to remain. Further research is required to establish the adequacy of the nutrient delivery of these AIO standard PN solutions.

## Implications

This study describes the process of both developing and implementing AIO standard PN solutions, which has not been documented before. We provide information on the actual composition of the standard PN and also the limitations, in order for other institutions to learn from both our success and mistakes.

## References

[1] Koletzko B., Goulet O., Hunt J., Krohn K., Shamir R., Parenteral Nutrition Guidelines Working Group, European Society for Clinical Nutrition and Metabolism, European Society of Paediatric Gastroenterology, Hepatology and Nutrition (ESPGHAN), European Society of Paediatric Research (ESPR) (2005). Guidelines on Paediatric Parenteral Nutrition of the European Society of Paediatric Gastroenterology, Hepatology and Nutrition (ESPGHAN) and the European Society for Clinical Nutrition and Metabolism (ESPEN), Supported by the European Society of Paediatric Research (ESPR). J. Pediatr. Gastroenterol. Nutr..

[2] Goulet O., Ruemmele F., Lacaille F., Colomb V. (2004). Irreversible intestinal failure. J. Pediatr. Gastroenterol. Nutr..

[3] Shulman R.J., Phillips S. (2003). Parenteral nutrition in infants and children. J. Pediatr. Gastroenterol. Nutr..

[4] Bonnabry P., Cingria L., Sadeghipour F., Ing H., Fonzo-Christe C., Pfister R. (2005). Use of a systematic risk analysis method to improve safety in the production of paediatric parenteral nutrition solutions. Qual. Saf. Health Care.

[5] Mirtallo J., Canada T., Johnson D., Kumpf V., Petersen C., Sacks G., Seres D., Guenter P. (2004). Safe practices for parenteral nutrition. JPEN J. Parenter. Enteral Nutr..

[6] Brown C.L., Garrison N.A., Hutchison A.A. (2007). Error reduction when prescribing neonatal parenteral nutrition. Am. J. Perinatol..

[7] Kochevar M., Guenter P., Holcombe B., Malone A., Mirtallo J. (2007). ASPEN statement on parenteral nutrition standardization. J. Parenter. Enteral Nutr..

[8] A Mixed Bag: An Enquiry into the Care of Hospital Patients Receiving Parenteral Nutrition. http://www.ncepod.org.uk/2010pn.htm.

[9] Fusch C., Bauer K., Bohles H.J., Jochum F., Koletzko B., Krawinkel M., Krohn K., Muhlebach S., Working Group for Developing The Guidelines for Parenteral Nutrition of The German Society for Nutritional Medicine (2009). Neonatology/Paediatrics—Guidelines on Parenteral Nutrition, Chapter 13. Ger. Med. Sci..

[10] Mackay M.W., Cash J., Farr F., Holley M., Jones K., Boehme S. (2009). Improving pediatric outcomes through intravenous and oral medication standardization. J. Pediatr. Pharmacol. Ther..

[11] Improving Practice and Reducing Risk in the Provision of Parenteral Nutrition for Neonates and Children. http://www.nppg.scot.nhs.uk/Minimising%20risk%20in%20PN%20for%20children.pdf.

[12] Klüttgens B.U., Sewell G.J., Nunn A.J. (2003). Current clinical practice in neonatal and paediatric parenteral nutrition in Europe. Eur. J. Hosp. Pharm..

[13] Bouchoud L., Fonzo-Christe C., Sadeghipour F., Bonnabry P. (2010). How standardised are paediatric parenteral nutrition formulations in Europe?. EJHP.

[14] Clayton P.T., Whitfield P., Iyer K. (1998). The role of phytosterols in the pathogenesis of liver complications of pediatric parenteral nutrition. Nutrition.

[15] Goulet O., Antebi H., Wolf C., Talbotec C., Alcindor L.G., Corriol O., Lamor M., Colomb-Jung V. (2010). A new intravenous fat emulsion containing soybean oil, medium-chain triglycerides, olive oil, and fish oil: A single-center, double-blind randomized study on efficacy and safety in pediatric patients receiving home parenteral nutrition. J. Parenter. Enteral Nutr..

[16] Diamond I.R., Pencharz P.B., Wales P.W. (2009). What is the current role for parenteral lipid emulsions containing omega-3 fatty acids in infants with short bowel syndrome?. Minerva Pediatr..

[17] Diamond I.R., Sterescu A., Pencharz P.B., Wales P.W. (2008). The rationale for the use of parenteral omega-3 lipids in children with short bowel syndrome and liver disease. Pediatr. Surg. Int..

[18] Krohn K., Babl J., Reiter K., Koletzko B. (2005). Parenteral nutrition with standard solutions in paediatric intensive care patients. Clin. Nutr..

[19] Rigo J., Marlowe M.L., Bonnot D., Senterre T., Lapillonne A., Kermorvant-Duchemin E., Hascoet J.M., Desandes R., Malfilatre G., Pladys P. (2012). Benefits of a new pediatric triple-chamber bag for parenteral nutrition in preterm infants. J. Pediatr. Gastroenterol. Nutr..

[20] Porras I., Cabello M.A., Oya Alvarez de M.B., Marin Pozo J.F., Garcia A.J., Llacer P.C. (2010). Assessment of standard parenteral nutrition in children (in Spanish). Nutr. Hosp..

[21] Narula P., Hartigan D., Puntis J.W. (2011). The frequency and importance of reported errors related to parenteral nutrition in a regional paediatric centre. e-SPEN.

[22] Bozzetti F., Arends J., Lundholm K., Micklewright A., Zurcher G., Muscaritoli M. (2009). ESPEN Guidelines on Parenteral Nutrition: Non-surgical oncology. Clin. Nutr..

[23] Wessel J.J., Kocoshis S.A. (2007). Nutritional management of infants with short bowel syndrome. Semin. Perinatol..

[24] Muhlebach S., Franken C., Stanga Z. Practical handling of AIO admixtures—Guidelines on Parenteral Nutrition, Chapter 10. Ger. Med. Sci..

[25] Seida J.C., Mager D.R., Hartling L., Vandermeer B., Turner J.M. (2013). Parenteral omega-3 fatty acid lipid emulsions for children with intestinal failure and other conditions: A systematic review. J. Parenter. Enteral Nutr..

[26] Muhammed R., Bremnerm R., Protheroe S., Johnson T., Holden C., Murphy M.S. (2012). Resolution of parenteral nutrition-associated jaundice on changing from a soybean oil emulsion to a complex mixed-lipid emulsion. J. Pediatr. Gastroenterol. Nutr..

[27] Skouroliakou M., Matthaiou C., Chiou A., Panagiotakos D., Gounaris A., Nunn T., Andrikopoulos N. (2008). Physicochemical stability of parenteral nutrition supplied as all-in-one for neonates. J. Parenter. Enteral Nutr..

[28] Wong T. (2012). Parenteral trace elements in children: Clinical aspects and dosage recommendations. Curr. Opin. Clin. Nutr. Metab. Care.

[29] Keady S., Morgan C., Ozzard A., Chauhan B. (2010). Effect of a neonatal standard aquaous parenteral nutrition formulation on aceptic unit capacity planning. e-SPEN.

[30] Gamsjager T., Brenner L., Schaden E., Sitzwohl C., Weinstabl C. (2009). Cost analysis of two approaches to parenteral nutrition in critically ill children. Pediatr. Crit. Care Med..

